# Comparative single-center study between modified laparoscopic radical hysterectomy and open radical hysterectomy for early-stage cervical cancer

**DOI:** 10.1186/s12957-022-02866-x

**Published:** 2022-12-12

**Authors:** Xuqing Li, Xueting Pei, Hongyan Li, Yan Wang, Youwei Zhou, Zhaolian Wei, Zongzhi Yin

**Affiliations:** 1 Department of Obstetrics and Gynecology, The First Affiliated Hospital of Anhui Medical University, Hefei, 230022 China; 2 NHC Key Laboratory of Study on Abnormal Gametes and Reproductive Tract (Anhui Medical University), Hefei, China; 3 Anhui Province Key Laboratory of Reproductive Health and Genetics, Hefei, Anhui China

**Keywords:** Modified laparoscopic radical hysterectomy, Open radical hysterectomy, Early-stage cervical cancer, Survival rate

## Abstract

**Background:**

Since the release of the LACC trial results in 2018, the safety of laparoscopic radical hysterectomy (LRH) for cervical cancer has received huge attention and heated discussion. We developed modified laparoscopic radical hysterectomy (MLRH) incorporating a series of measures to prevent tumor spillage, which has been performed in our center since 2015.

**Objective:**

Present study retrospectively analyzed relevant indicators of MLRH and evaluated disease-free survival (DFS) primarily in the treatment of early cervical cancer compared with open surgery.

**Methods:**

Patients with 2014 International Federation of Gynecology and Obstetrics clinical stages 1B1 and 2A1 cervical cancer who underwent radical hysterectomy in the gynecological department of our hospital from October 2015 to June 2018 were enrolled retrospectively in this study. Patients were divided into two groups based on the surgical procedure: open radical hysterectomy (ORH) group (*n* = 336) and MLRH group (*n* = 302). Clinical characteristics, surgical indices, and survival prognosis were analyzed, including 2.5-year overall survival (OS) rate, 2.5-year DFS rate, recurrence rate, and recurrence pattern.

**Results:**

Compared to the ORH group, the MLRH group exhibited a longer operative time, longer normal bladder function recovery time, less intraoperative blood loss volume, and more harvested pelvic lymph nodes (*P* < 0.05). No significant differences were observed in postoperative complications, the 2.5-year OS, 2.5-year DFS, and recurrence rate between the two groups (*P* > 0.05); however, the recurrence pattern was significantly different (*P* < 0.05). The MLRH group mainly exhibited local single metastasis (7/11), whereas the ORH group mainly exhibited distant multiple metastases (14/16). Stratified analysis revealed that overall survival rate was higher in the MLRH group than in the ORH group in patients with stage 1B1 and middle invasion (*P* < 0.05).

**Conclusion:**

MLRH does not show a survival disadvantage in the treatment of early-stage cervical cancer when compared with open surgery. In addition, MLRH shows a survival advantage in patients with stage 1B1 and middle 1/3 invasion. Considering this is a retrospective study, further prospective study is necessary for more sufficient data support.

**Trial registration:**

Present research is a retrospective study. The study had retrospectively registered on Chinese Clinical Trial Registry (http://www.chictr.org.cn/), and the registered number is ChiCTR1900026306.

**Supplementary Information:**

The online version contains supplementary material available at 10.1186/s12957-022-02866-x.

## Background

Cervical cancer ranks fourth in the global incidence of malignant tumors in women, and 85% of cases occur in developing countries, which takes one of the leading contributors to cancer-related deaths in women [1]. The incidence of cervical cancer in China is increasing, with 98,900 new cases and 30,500 deaths reported in 2015 [2]. Previous guidelines [3, 4] have indicated that both open and minimally invasive approaches are useful for the surgical treatment of early-stage cervical cancer, which was defined as tumors 4 cm or less that are confined to the cervix by 2014 International Federation of Gynecology and Obstetrics (FIGO) [5]. Retrospective studies and meta-analyses have reported that blood loss volume, hospital stay, and postoperative complication rates of the minimally invasive approach are lower than those of open surgery, whereas the 5-year disease-free survival (DFS) and overall survival (OS) rates were similar between the approaches [6–9]. A meta-analysis by Cao et al. [10] of 2922 patients with early-stage cervical cancer revealed no significant differences in DFS, OS, and recurrence rates between the two groups.

In contrast, a multicenter randomized controlled trial named the Laparoscopic Approach to Cervical Cancer (LACC) trial by Ramirez et al. [11] in 2018 reported that the 4.5-year DFS and OS rates of minimally invasive surgery were lower, and the recurrence rate was higher than that of laparotomy in patients with early-stage cervical cancer. A retrospective analysis by Melamed et al. [12] reported that the mortality rate was higher in the minimally invasive group than in the open group. According to the SEER database, the 4-year survival rate of minimally invasive surgery decreased at an annual rate of 0.8% after 2006. The application of minimally invasive surgery in the treatment of early-stage cervical cancer has generated substantial controversy worldwide.

Notably, there are some limitations of the LACC trial which included bias relevant to research center and missing elaboration on possible factors contributing to the results. Therefore, the advantages and drawbacks of laparoscopic radical hysterectomy (LRH) should be reconsidered. At present, the international classical operation of LRH requires a uterine manipulator to control the uterus. Common surgical procedures include resecting the anterior lobe of the bladder cervical ligament to separate the ureter, resecting the paracervical main sacral ligament, and finally cutting off the vagina in the pelvic cavity under laparoscopic vision. Most scholars have suggested that these procedures lead to tumor spillage and dissemination, which may be a critical reason underpinning the inferior survival outcomes in the minimally invasive approach. When laparoscopic surgery was first applied to colon cancer, potential peritoneal dissemination or metastasis raised substantial attention [13]. Peritoneal dissemination may be due to tumor perforation, operational tumor extrusion, or carbon dioxide (CO_2_) pneumoperitoneum [14], which can be prevented by improved measures [15].

Compared to classic LRH, modified laparoscopic radical hysterectomy (MLRH) incorporates a series of procedures to prevent tumor spillage, such as avoiding the use of a uterine manipulator [16], initial resection of the paracervical main sacral ligament, cutting off the vagina following stapler closure [17], and the use of double-distilled water to flush the wound throughout the operation [18]. In order to further evaluate the effects of MLRH, we retrospectively analyzed the surgical outcomes and survival prognosis especially DFS of patients subjected to different operative approaches in our center.

## Methods

### Study population

The sample size is based on an observed DFS rate from the LACC trails. Assume that the 2.5-year DFS rate for patients who underwent open surgery is 97.1%, consistent with the data in LACC trial. To show that the 2.5-year DFS rate in laparoscopic surgery group is not worse than 91.2%, the study needs to recruit at least 244 people for each group with a two-sided level of significance (*α*) of 5% and a power (1-β) of 80%. This power calculation was performed with PASS 15.0 (Power Analysis and Sample Size, copyright 2017, NCSS, LLC).

**Fig. 1:**
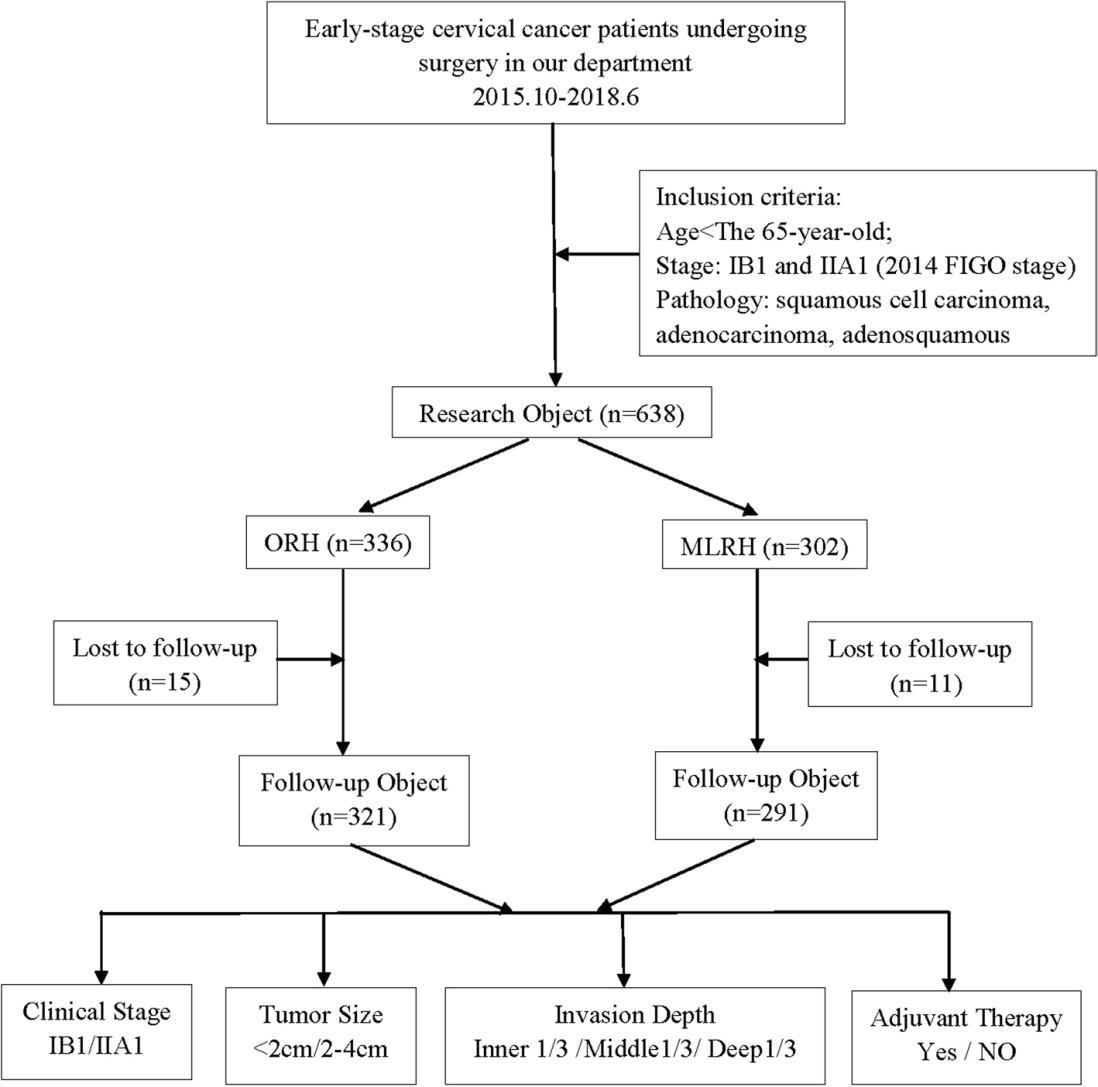
Patient inclusion and grouping process. ORH, open radical hysterectomy; MLRH, modified laparoscopic radical hysterectomy

A total of 638 patients with clinical early-stage cervical cancer undergoing radical hysterectomy between October 2015 and June 2018 were included. Patients were divided into the ORH group (*n* = 336) or MLRH group (*n* = 302). The patients selected the surgical approach after professional consultation. The study was approved by the local ethics committee and conducted in accordance with the Declaration of Helsinki as revised in 2013. The specific inclusion and grouping process are presented in the Fig. 1.

Inclusion criteria were as follows: (1) age ≤ 65 years; (2) clinical stages: IB1 and IIA1 (2014 FIGO staging system); and (3) pathological type: squamous cell carcinoma, adenocarcinoma, and adenosquamous carcinoma. Exclusion criteria were as follows: (1) histologic types: neuroendocrine carcinoma, clear cell carcinoma, serous carcinoma, small cell carcinoma, or minimal deviation adenocarcinoma, (2) neoadjuvant chemotherapy or radiotherapy before surgery, and (3) severe systemic underlying diseases, immune diseases, mental illness, or other malignancies.

### Operative procedure

Based on Querleu and Morrow’s classification, all patients underwent primary type C2 hysterectomy and pelvic lymph node dissection (with or without para-aortic lymph node sampling). All surgeries were performed by three doctors with rich experience in radical hysterectomy. And a quality control team in our center was established to reach a consensus on operation.

### ORH procedure

After induction of general anesthesia, patients were placed in the supine position. A median longitudinal incision was made from the symphysis pubis to 4 cm above the navel in the abdomen. The open pelvic cavity was explored, pelvic lymph node was dissected, and extensive hysterectomy was performed.

### MLRH procedure

Patients adopted the bladder lithotomy position, and five puncture holes were selected after establishing artificial CO_2_ pneumoperitoneum. A series of measures were incorporated to prevent tumor spillage based on general laparoscopic procedures: (1) avoiding the use of a uterine manipulator by using an acutenaculum to push and pull the suture for manipulating the uterus through the fifth puncture, (2) immediately bagging the whole pelvic lymph node after resection, (3) resecting the paracervical main sacral ligament and anterior lobe of the bladder cervical ligament first after lymph node dissection, (4) cutting off the vagina after stapler closure and removing the uterus after vaginal pouch suture, (5) flushing the open pelvic cavity repeatedly with double-distilled water throughout the operation, and (6) reducing the intraoperative pneumoperitoneum pressure by maintaining the pneumoperitoneum pressure at 11 mmHg.

### Follow-up

All patients were followed up every 3 months in the first 2 years, every 6 months in the next 3–5 years, and then once a year. All patients (except those who were deceased) were followed up until December 31, 2020. Follow-up items included vaginal cytology, laboratory examination, and imaging examination. Recurrent cases were detected and diagnosed timely according to symptoms, signs, and auxiliary examination results.

### Research variables

General clinical characteristics included age, body mass index (BMI), clinical stage, histological grading, pathological type, tumor size, intermediate-risk and high-risk factors, and adjuvant therapy. Clinical stage referred to the FIGO staging method. To determine tumor size, preoperative evaluation of the maximum diameter of the tumor measured according to gynecological examination and pelvic magnetic resonance imaging was performed. Intermediate-risk factors included tumor diameter, invasion depth, and vascular tumor thrombus positivity. High-risk factors included positive results for para-uterine and vaginal margin and lymph node metastasis. Adjuvant treatments included radiotherapy, chemotherapy, or concurrent chemoradiation therapy (CCRT) [19], which referred to the Sedlis standard.

### Operative outcomes

Operative outcomes included the operative time, blood loss volume, number of lymph nodes harvested, normal bladder function recovery time, and postoperative complications. Normal bladder function recovery time was determined based on residual urine < 50 ml by ultrasonography. Postoperative complications included urinary system injury, intestinal obstruction, peritonitis, thrombus, perineum and lower extremity edema, symptomatic lymphatic cyst, and delayed incision healing. Survival prognosis included the DFS rate, OS rate, recurrence rate, and recurrence pattern. Further analysis was completed by stratification of different factors.

### Statistical analysis

Continuous normal distribution variables were expressed as mean ± standard deviation (*SD*) and compared by *t*-test. Continuous non-normal distribution variables were expressed as median (*P*_25_-*P*_75_) and compared by Mann-Whitney *U*-test. The Mann-Whitney test was used for comparing median values and Student’s *t*-test for comparing mean values. Categorical variables were expressed as frequency (percentage) and compared by Pearson chi-square test or Fisher’s exact test. Survival outcomes were compared using Kaplan-Meier analysis with the log-rank test. Statistical analyses were performed using SPSS statistical software (version 25.0). *P* < 0.05 was considered statistically significant.

## Results

### Clinical characteristics

Mean age of patients was higher in the ORH group than in the MLRH group (mean: 48.55 years versus [vs.] 46.92 years, respectively; *P* = 0.016). Mean BMI was similar between the groups (ORH and MLRH: mean: 23.39 kg/m^2^ and 23.20 kg/m^2^, respectively: *P* = 0.442). Significant between-group differences were observed in clinical stage (*P* < 0.001) and invasion depth (*P* = 0.001). No significant differences were observed in histological grade, pathological type, vascular tumor emboli-positive rate, lymph node metastasis rate, and adjuvant therapy (*P* < 0.05). Specific results are presented in Table 1.

**Table 1:** Clinical characteristics of the patients

Characteristic	ORH	MLRH	*p*-value
Total	336	302	
Patients lost to follow-up (*n*)	15	11	
Patients followed up with (*n*)	321	291	0.016
Age (y)	48.55 ± 8.51	46.92 ± 8.10	
BMI (kg/m^2^)	23.39 ± 2.98	23.20 ± 3.15	0.442
Histological grading (%)			0.146
G1	23 (7.2)	45 (15.5)	
G2	173 (53.9)	136 (46.7)	
G3	125 (38.9)	110 (37.8)	
Pathological type (%)			0.700
Squamous cell carcinoma	262 (81.6)	233 (80.1)	
Adenocarcinoma	55 (17.1)	52 (17.9)	
Adenosquamous carcinoma	4 (1.2)	6 (2.1)	
FIGO stage (%)			< 0.001
IB1	265 (82.6)	269 (92.4)	
IIA1	56 (17.4)	22 (7.6)	
Tumor diameter (%)			0.079
< 2 cm	87 (27.1)	98 (33.7)	
2~4 cm	234 (72.9)	193 (66.3)	
Invasion depth (%)			0.001
Inner 1/3	119 (37.1)	142 (48.8)	
Middle 1/3	116 (36.1)	97 (33.3)	
Deep 1/3	86 (26.8)	52 (17.9)	
Vascular tumor embolus (%)			0.325
Negative	243 (75.7)	230 (79.0)	
Positive	78 (24.3)	61 (21.0)	
Lymph node metastasis (%)			0.143
Negative	258 (80.4)	247 (84.9)	
Positive	63 (19.6)	44 (15.1)	
Adjuvant therapy (%)			0.058
None	163 (50.8)	179 (61.5)	
Radiotherapy	35 (10.9)	28 (9.6)	
Chemotherapy	22 (6.9)	14 (4.8)	
CCRT	101 (31.5)	70 (24.1)	

*FIGO* 2014 International Federation of Gynecology and Obstetrics, *CCRT* concurrent chemoradiation therapy, *ORH* open radical hysterectomy, *MLRH* modified laparoscopic radical hysterectomy

### Operative outcomes

Compared with the ORH group, the MLRH group exhibited a longer operative time (median: 180 min vs. 200 min; *P* < 0.001), longer normal bladder function recovery time (median: 14 days vs 14 days; mean: 14.61 days vs. 15.33 days; *P* = 0.034), less blood loss volume (median: 150 mL vs. 100 mL; *P* < 0.001), and more harvested lymph nodes (median: 18 vs. 20; *P* = 0.005). No significant between-group difference was noted in the incidence of postoperative complications (ORH and MLRH: 15.0% and 10.3%, respectively; *P* = 0.085) (Table 2).

**Table 2:** Operating outcomes of patients

Characteristic	ORH	MLRH	*p*-value
Operative time (min)	180 (150–220)	200 (180–240)	< 0.001
Blood loss volume (mL)	150 (100–200)	100 (50–100)	< 0.001
Lymph nodes harvested (*n*)	18 (14–22)	20 (15–24)	0.005
Normal bladder function recovery time (d)^a^	14 (13–15)	14 (13–15)	0.034
Postoperative complications (%)			0.085
None	273 (85.0)	261 (89.7)	
Yes	48 (15.0)	30 (10.3)	
Urinary damage	5 (1.6)	4 (1.4)	1.000
Intestinal obstruction	9 (2.8)	7 (2.4)	0.758
Thrombus	3 (0.9)	2 (0.7)	1.000
Symptomatic lymphatic cyst	10 (3.1)	5 (1.7)	0.264
Delayed incision healing	7 (2.2)	3 (1.0)	0.423
Perineal and lower limb edema	8 (2.5)	6 (2.1)	0.722
Peritonitis	14 (4.4)	11 (3.8)	0.717

*ORH* open radical hysterectomy, *MLRH* modified laparoscopic radical hysterectomy

^a^The mean normal bladder function recovery time in the ORH group and MLRH group was 14.61 day and 15.33 day, respectively

### The survival outcome and recurrent pattern

No significant differences were observed in the 2.5-year OS, DFS, and recurrence rates (*P* > 0.05) between groups. Recurrence pattern was significantly different between the groups (*P* = 0.019). The ORH group mainly exhibited distant multiple metastases (14/16), whereas the MLRH group mainly exhibited local single metastasis (7/11). Specific data are presented in Table 3.

**Table 3:** Survival and recurrence outcome of patients

Characteristic	ORH	MLRH	*p*-value
OS rate (%)*	313 (97.5)	289 (99.3)	0.150
DFS rate (%)	310 (96.6)	281 (96.6)	0.995
Recurrence rate (%)	11 (3.4)	10 (3.4)	0.995
Recurrence pattern			0.019
Local single (%)	2 (12.5)	7 (63.6)	
Distant multiple (%)	14 (87.5)	4 (36.4)	

*ORH* open radical hysterectomy, *MLRH* modified laparoscopic radical hysterectomy, *OS* overall survival, *DFS*, disease-free survival

### Stratified comparison of survival prognosis by different surgical approach

The prognosis of the ORH and MLRH groups was stratified and compared based on factors such as the clinical stage, tumor size, invasion depth, and adjuvant treatment. Results showed that in patients with stage IB1 and middle invasion, the OS rate was higher in the MLRH group than in the ORH group (99.6% vs 97.7%, *P* = 0.011, Additional file 1; 100.0% vs 98.3%, *P* = 0.043, Additional file 3,). There was no significant difference in the DFS rate and OS rate between the ORH group and MLRH group for all other stratifications. Specific results are shown in Additional files 1, 2, 3, and 4.

## Discussion

The surgical scope of patients in both groups was based on the standard of C-type hysterectomy in Q-M classification. The paracervical and vaginal tissues were removed according to the anatomical markers of C-type hysterectomy. The results showed that operative time was longer, blood volume was lower, and number of lymph nodes harvested was higher in the MLRH group than in the ORH group, which is consistent with findings of previous reports [20, 21]. The microscopic multi-angle vision of the laparoscope enables clearer exposure of tissue anatomy, which is conducive to a more meticulous operation and more satisfactory operation quality control effect. The recovery time of normal bladder function was longer in the MLRH group than in the ORH group, which could be due to electrothermal conductive nerve injury caused by energy instruments [22]. No significant difference between group was noted in the incidence of postoperative complications, consistent with the results of the LACC test [11].

The LACC trial demonstrated that the 3-year DFS rates of the minimally invasive and open groups were 91.2% and 97.1%, respectively (95% *CI* 1.63~8.58, *P* < 0.05), whereas the 3-year OS rates were 93.8% and 99.0%, respectively (95% *CI* 1.77~20.30, *P* < 0.05) [11]. This indicated that minimally invasive radical hysterectomy was associated with lower DFS and OS in patients with early-stage cervical cancer. But our results revealed no significant differences in the 2.5-year OS rate, DFS rate, and recurrence rate between the MLRH and ORH groups. This may benefit from additional procedures for preventing tumor cell spillage and micrometastasis in MLRH [23]. Kanao [24] compared the outcomes of laparoscopic surgery with the no-look no-touch technique to those of open surgery in the treatment of early-stage cervical cancer, and no significant between-group differences were noted in the 2.5-year DFS (94.4% vs. 90.9%, *P* = 0.591) and OS (100% vs. 96.5%, *P* = 0.188), which is consistent with our results.

In the LACC trial, the recurrence pattern of the laparoscopic group manifested predominantly as multiple pelvic recurrence, distinct to that of the open group [11]. However, our study demonstrated that there was more pelvic local recurrence in the MLRH group than in the ORH group and more distant multiple recurrences in the ORH group than in the MLRH group. Possible reasons for this include the improved procedures of laparoscopic surgery having a definite effect on the prevention of tumor cell spillage and repeated extrusion of cervical lesions during open surgery leading to tumor micrometastasis [25]. Particularly, when the cervical tumor is difficult to expose, the cervix will inevitably be extruded during open operation. In the MLRH, we exposed the operating space fully by suspension of the uterus, which ensured that the tumor was completely free of touch and extrusion.

The clinical stage of patients with cervical cancer in our study was determined based on the 2014 FIGO staging criteria [26]. IB stages are divided into stage IB1 (tumor diameter ≤ 2 cm), stage IB2 (tumor diameter 2–4 cm), and stage IB3 (tumor diameter > 4 cm) based on the latest 2018 FIGO stage [27]. Tumor size is closely related to the survival prognosis of cervical cancer [28]. At the 2018 Annual Meeting of the American Clinical Oncologic Society, scholars proposed that OS was not lower for laparoscopic surgery than open surgery in patients with cervical cancer of diameter < 2 cm [29]. Kim et al. [30, 31] reported similar conclusions in their recently published studies. To eliminate the effects of adjuvant therapy, Paik et al. [32] selected IB1 cervical cancer patients without adjuvant treatment for retrospective analysis and reported that DFS was significantly lower in minimally invasive surgery than in open surgery.

Accordingly, our data were further stratified based on clinical stage, tumor diameter, and adjuvant treatment. For patients with stage IB1 and middle invasion, OS rate was obviously higher in the MLRH group than in the ORH group. The chance of paracervical micrometastases may increase in this part of patients, so satisfactory and reliable paracervical resection range is particularly important. The results showed that more cases with stage 2A1 and deep infiltration were selected in ORH group than MLRH group. This subset of patients may indeed be associated with distant metastasis. The advantage of laparoscopic microscope magnification, precise paracervical ligament resection, and avoidance of touch contribute obviously to the radicality of paracervical resection, which is the probable cause of the OS difference. There was no deliberate tendency to laparoscopic surgery in IB1 patients with moderate risk factors. All the risk factors were determined by postoperative pathological results.

The chance of parametrial micrometastases is reduced in patients with superficial invasion. Therefore, smaller paracervical resection range may also achieve therapeutic effect. Correspondingly, many studies have reported that there was no difference on the prognosis in very early cervical cancer between minimally invasive and open surgery approach [29–31]. For the patients with later stage and more severe local tumor lesions, the chance of postoperative supplemental radiotherapy increased, which resulted in the increase of confounding influencing factors on the prognosis of patients. Therefore, it is impossible to evaluate the solitary influence of surgical approach on the prognosis, reliably. In the present study, no significant differences were observed in DFS and OS between the two subgroups with a tumor diameter 2–4 cm and who received adjuvant therapy. However, the *P*-value of OS approximated 0.05 (*P* = 0.056; *P* = 0.068). Larger samples are required for confirmation in the future.

Cervical cancer with pelvic and/or para-aortic lymph node metastases is classified as stage 3C based on the latest 2018 FIGO stage, regardless of tumor size and extent of spread. Some scholars [33] proposed that the number of metastatic pelvic lymph nodes may be related to the prognosis of cervical cancer with stage 3C1, especially the metastatic number exceeds the critical value of 2. This study mainly investigated the influence of different surgical approaches on the survival prognosis of early cervical cancer. The proportion of patients with metastatic pelvic lymph nodes in our study was small, so further stratified analysis was not conducted.

### Strengths

In the present study, patients in the MLRH group were treated with novel surgical methods, adding a series of innovative procedures to prevent tumor extravasation, and the surgeries were performed by the same gynecological team, thereby avoiding the influence of surgeons’ learning curve on the results [34, 35].

### Limitations

This was a retrospective study, and the included subjects were not randomized, potentially leading to sample selection bias. For patients who received surgical treatment only from one center, the influence of institutional heterogeneity, especially differences in the surgical operation on the results, could not be analyzed [36]. The surgical team may have a tendency in the process of communicating with patients. As a result, the random grouping cannot be absolute guaranteed. At the same time, the follow-up time was relatively short. MRLH has mainly been performed since 2015. The median follow-up time was 42 months (30–62 months), which could be extended to more than 5 years later.

## Conclusion

MLRH follows the tumor-free principle strictly and incorporates a series of measures to prevent tumor spillage at the same time. Compared with ORH, there was no significant difference in the survival outcome of patients with early-stage cervical cancer by MLRH.

In consideration of better surgery quality control and more accessible promotion, the clinical application prospect of MLRH in the treatment of early-stage cervical cancer is expectant. Further prospective randomized controlled studies can be implemented to obtain more powerful evidence support in the future.

## Supplementary Information

**Additional file 1.** Comparison of survival prognosis by surgical approach (clinical stage). Clinical stage: IB1 (A: DFS; B: OS); IIA1 (C: DFS; D: OS). ORH, open radical hysterectomy; MLRH, modified laparoscopic radical hysterectomy; OS, overall survival; DFS, disease-free survival; PFS, progression-free survival.

**Additional file 2.** Comparison of survival prognosis by surgical approach (tumor diameter). Tumor diameter: < 2 cm (A: DFS; B: OS); 2–4 cm (C: DFS; D: OS). ORH, open radical hysterectomy; MLRH, modified laparoscopic radical hysterectomy; OS, overall survival; DFS, disease-free survival; PFS, progression-free survival.

**Additional file 3.** Comparison of survival prognosis by surgical approach (invasion depth). Invasion depth: Inner 1/3 (A: DFS; B: OS); Middle 1/3 (C: DFS; D: OS); Deep 1/3 (E: DFS; F: OS). ORH, open radical hysterectomy; MLRH, modified laparoscopic radical hysterectomy; OS, overall survival; DFS, disease-free survival; PFS, progression-free survival.

**Additional file 4.** Comparison of survival prognosis by surgical approach (adjuvant therapy). Adjuvant therapy: AB without adjuvant therapy (A: DFS, B: OS), CD with adjuvant therapy (C: DFS, D: OS). ORH, open radical hysterectomy; MLRH, modified laparoscopic radical hysterectomy; OS, overall survival; DFS, disease-free survival; PFS, progression-free survival.

## Data Availability

The datasets used and/or analyzed during the current study are available from the corresponding author on reasonable request.

## Abbreviations

MLRH: Modified laparoscopic radical hysterectomy
FIGO: International Federation of Gynecology and Obstetrics
ORH: Open radical hysterectomy
OS: Overall survival
DFS: Disease-free survival
LACC: Laparoscopic approach to cervical cancer
LRH: Laparoscopic radical hysterectomy
BMI: Body mass index
